# Saccharibacteria as Organic Carbon Sinks in Hydrocarbon-Fueled Communities

**DOI:** 10.3389/fmicb.2020.587782

**Published:** 2020-12-23

**Authors:** Perla Abigail Figueroa-Gonzalez, Till L. V. Bornemann, Panagiotis S. Adam, Julia Plewka, Fruzsina Révész, Christian A. von Hagen, András Táncsics, Alexander J. Probst

**Affiliations:** ^1^Group for Aquatic Microbial Ecology, Environmental Microbiology and Biotechnology, Faculty of Chemistry, University of Duisburg-Essen, Essen, Germany; ^2^Regional University Center of Excellence in Environmental Industry, Szent István University, Gödöllõ, Hungary; ^3^Department of Environmental Protection and Environmental Safety, Szent István University, Gödöllõ, Hungary

**Keywords:** hydrocarbon degradation, symbionts, groundwater, genome-resolved metagenomics, enrichment cultures

## Abstract

Organisms of the candidate phylum Saccharibacteria have frequently been detected as active members of hydrocarbon degrading communities, yet their actual role in hydrocarbon degradation remained unclear. Here, we analyzed three enrichment cultures of hydrocarbon-amended groundwater samples using genome-resolved metagenomics to unravel the metabolic potential of indigenous Saccharibacteria. Community profiling based on ribosomal proteins revealed high variation in the enrichment cultures suggesting little reproducibility although identical cultivation conditions were applied. Only 17.5 and 12.5% of the community members were shared between the three enrichment cultures based on ribosomal protein clustering and read mapping of reconstructed genomes, respectively. In one enrichment, two Saccharibacteria strains dominated the community with 16.6% in relative abundance and we were able to recover near-complete genomes for each of them. A detailed analysis of their limited metabolism revealed the capacity for peptide degradation, lactate fermentation from various hexoses, and suggests a scavenging lifestyle with external retrieval of molecular building blocks. In contrast to previous studies suggesting that Saccharibacteria are directly involved in hydrocarbon degradation, our analyses provide evidence that these organisms can be highly abundant scavengers acting rather as organic carbon sinks than hydrocarbon degraders in these communities.

## Introduction

Groundwater systems represent approximately 30% of the global freshwater reservoirs, with their main uses being irrigation systems and water supply for human consumption (Foster and Chilton, 2003). These freshwater systems are at risk for anthropogenic contamination like release of toxic chemicals from municipal waste landfills, abandoned production facilities or accidental hydrocarbon spills (Schwarzenbach et al., 2010). Petroleum hydrocarbons, composed of complex molecules such as aliphatic alkanes, benzene, toluene, ethylbenzene, and xylene (BTEX), and polycyclic aromatic hydrocarbons (PAHs), pose a great threat to human health and the environment due to their toxic, mutagenic, and carcinogenic potential as well as their high bioaccumulation potential (Anjum et al., 2017; Mahmoud and Bagy, 2019). It is estimated that at least 1.5 billion tons of petroleum oil are yearly transported (Schwarzenbach et al., 2010), and leakage to the environment occurs either by accident or simply due to the handling of petroleum (e.g., extraction or refinement processes), with estimates between 1.7 and 1.8 million metric tons of crude oil reaching water bodies around the world (Anjum et al., 2017). Consequently, contamination of groundwater aquifers by petroleum represents a great ecological and societal problem (Mahmoud and Bagy, 2019).

Alkanes make up a large fraction of crude oil (sometimes >50%) and are chemically inert molecules. Their low reactivity, which translates to low microbial bioavailability, is part of what makes them a severe ecological problem when released into the environment (Singh et al., 2012). Microbial degradation processes of these compounds are dependent on the presence of electron acceptors. Oxygen serves as the electron acceptor for oxic bioremediation, while nitrate and sulfate can be the electron acceptors for the anoxic counterpart (Farhadian et al., 2008). In general, optimal degradation of hydrocarbons mainly occurs under aerobic conditions, and a key step is the incorporation of oxygen by oxygenases. The overall degradation yields intermediates of the tricarboxylic acid (TCA) cycle, which can be further used for cell growth and maintenance (Berthe-Corti and Fetzner, 2002; Singh et al., 2012). There are a plethora of bacteria that have been identified to be able to degrade hydrocarbons (Steiert et al., 1987; Chaillan et al., 2004; Throne-Holst et al., 2007; Abdel-Megeed et al., 2010; Singh et al., 2012; Prince et al., 2019). Interestingly, some studies point to the presence of members of the Candidate Phyla Radiation (CPR) in petroleum contaminated sites (Chong et al., 2018; Salam et al., 2018; Révész et al., 2020b). Luo et al. (2009) used stable isotope probing (SIP) for the identification of a member of the phylum TM7 (now widely known as Saccharibacteria) in ^13^C_6_-toluene-amended enrichment cultures. These enrichments were inoculated with agricultural soil from a field, which had been in contact with biosolids. After terminal restriction fragment length polymorphism (TRFLP), they found that one fragment of 394 bp was enriched in the ^13^C-toluene fraction, and the signal increased over incubation time. 16S rRNA gene analyses identified the fragment to belong to a member of TM7. Their findings point to a potential involvement of TM7 in toluene degradation, but no further metabolic analysis had been performed. In a similar SIP study, this time in microcosms amended with ^13^C_6_ labeled benzene, the DNA of a member of TM7 was also identified to become labeled after incubation. The microcosms were inoculated from three soil samples, one of these was collected from a gasoline contaminated site and had been exposed to benzene, while the other two were obtained from agricultural sites and had no previous exposure to benzene but had been in contact with biosolids from a wastewater treatment plant. The degraders detected from the gasoline contaminated site were members of *Polaromonas* sp. and *Acidobacterium*, while TM7 was identified in the enrichments from one of the agricultural soil samples (Xie et al., 2011). Comparison of the 16S rRNA gene of the TM7 identified in both the toluene and benzene enrichment cultures showed that they diverge in their nucleotide identity, and points to a potential for members of this phylum to be involved in hydrocarbon degradation (Luo et al., 2009; Xie et al., 2011). No in-depth metabolic analysis of the Saccharibacteria in the two above mentioned studies was performed and hence their potential role in the microbial community has not been elucidated. Thus, the question arises if Saccharibacteria are involved in hydrocarbon degradation and how their metabolic potential is structured.

Saccharibacteria belong to the so-called CPR, a phylogenetic cluster of bacteria that comprises about 25% of this domain (Brown et al., 2015; Hug et al., 2016) and contains at least 73 phyla (Bor et al., 2020). Most of their members remain uncultivated, but they have been identified and studied through cultivation-independent approaches, such as metagenomics and 16S rRNA gene surveys. Genomic analyses have shown that they frequently lack reactions or even complete biosynthetic pathways for important cellular building blocks (e.g., fatty acids, proteins, and nucleotides) (Castelle et al., 2018), although some members have the potential for fermentation and synthesis of certain co-factors (Castelle et al., 2018). Support for their limited metabolism stems from genomic sequences and very few cultivated representatives of the phylum Saccharibacteria. He et al. (2015) reported the culture of a Saccharibacteria strain, which they named TM7× and have proposed to rename as *Nanosynbacter lyticus* (McLean et al., 2018). They were able to grow the Saccharibacteria with its host *Actinomyces odontolyticus* strain XH001, from samples of the oral cavity of humans, and identified it as a coccus-shaped epibiont with a cell size of 200–300 nm and a genome of 705 kbp. Interestingly, *N. lyticus* shows a parasitic behavior toward *A. odontolyticus*, when grown in low nutrient media, a trait not shown when grown in high nutrient media. It also shows a strong dependence on the host, since it has been reported that it is unable to grow in its absence (He et al., 2015). Studies on the host dynamics of TM7× with *A. odontolyticus* XH001 have shown that *N. lyticus* is capable of killing its host cells, albeit over time XH001 can adapt to the presence of the epibiont, in a stable symbiotic relationship (Bor et al., 2018). Furthermore, TM7× seems to be host-dependent on strains from the genus *Actinomyces* and some of these host strains can exhibit a growth/crash/recovery phase, depending on the initial dosage of TM7× cells it is exposed to Utter et al. (2020). Cross and collaborators successfully isolated and cultivated three different Saccharibacteria strains, including one closely related to TM7× (within the order *Candidatus* Teamsevenales), from human oral samples (Cross et al., 2019). Despite these cultivated strains, the majority of CPR remain uncultivated and further studies are still needed to understand their metabolism and symbiotic partnerships.

In this study, we aimed at filling the gap about the metabolic potential of Saccharibacteria that enriched during hydrocarbon amendment of groundwater samples and challenged the above-mentioned conclusion that Saccharibacteria are directly involved in hydrocarbon degradation. We established enrichment cultures from gasoline-contaminated groundwater and amended them with diesel mixtures to enrich for Saccharibacteria. We resolved the genomes of Saccharibacteria using metagenomic sequencing and identified their metabolic role in the respective community.

## Materials and Methods

### Enrichment Cultures

Oxic enrichment cultures were set up in triplicates, named AER1, AER2, and AER3, using 45 mL of freshwater media dispensed in 100 mL crimp-sealed serum bottles (Révész et al., 2020a). Biofilm samples were collected from a gasoline contaminated groundwater well (Benedek et al., 2016), of which 1 g (wet weight) was mixed with 99 mL of physiological saline solution. 5 mL of this solution were used as inoculum for each enrichment. Carbon and energy sources were added in the form of 20 ppm diesel fuel/crude oil mixture (3:2 v/v). Both crude oil and pure (additive-free) diesel fuel were obtained from the Hungarian Oil and Gas Plc. (MOL Plc.). Dissolved oxygen concentration in the bottles was monitored non-invasively by Fibox 3 trace v3 fiber optic oxygen meter with PSt3 sensor spots (PreSens). To maintain oxic conditions (∼7–8 mg/L), oxygen was replenished in the enrichments by flushing the liquid phase with sterile air under aseptic conditions once in every 24 h. Incubation was done in a rotary incubator at 28°C, 150 rpm. 5 mL of each enrichment was transferred to new media every week for five consecutive weeks. Cells were harvested by centrifugation (4°C, 2360 × *g*, 10 min), and DNA extraction was done using the DNeasy UltraClean Microbial Kit (Qiagen).

### Genome-Resolved Metagenomics

Sequencing was performed with an Illumina MiSeq sequencer, using the MiSeq Reagent Kit v2 (500-cycles) to generate paired-end read library (2 × 250 nucleotides) with 600–800 bps insert size by SeqOmics Biotechnology Ltd. (Mórahalom, Hungary). Quality check of raw reads was performed using BBduk (v. 37.09)^1^ and SICKLE (Joshi and Fass, 2011). Reads were assembled and scaffolded using metaSPAdes (v 3.13) (Nurk et al., 2017). Gene prediction, for scaffolds larger than 1 kb, was done using Prodigal in meta mode (Hyatt et al., 2010), and annotated using DIAMOND blast (Buchfink et al., 2014) against UniRef100 (Suzek et al., 2007). 16S rRNA gene sequences were predicted as described by Brown et al. (2015) and annotated against SILVA 132 (Quast et al., 2013). A consensus taxonomy for each scaffold was calculated using the taxonomy of the annotated genes (Bornemann et al., 2020). After mapping reads to scaffolds using Bowtie2 (mode -sensitive) (Langmead and Salzberg, 2012), average scaffold coverage, GC content, and length were calculated. Binning of samples was done using tetranucleotide-based Emergent Self-Organizing Maps (ESOM) (Dick et al., 2009). Obtained bins were further cleaned using GC content, coverage, and taxonomy of the scaffolds (Bornemann et al., 2020). Completeness of bins was estimated based on the presence of 51 universal bacterial single copy genes (SCG) (Probst et al., 2017) and confirmed using CheckM (Parks et al., 2015). Only bins with ≥70% estimated completeness and ≤10% contamination were used for further analyses. To calculate the phylogenetic position of the draft genomes we followed the approach described in Hug and collaborators. In brief, 16 ribosomal proteins (L2, L3, L4, L5, L6, L14, L15, L16, L18, L22, L24, S3, S8, S10, S17, and S19) were extracted (Hug et al., 2016) and aligned using Multiple Sequence Comparison by Log-Expectation (MUSCLE) (v3.8.31) (Edgar, 2004) with default parameters. The aligned sequences were end-trimmed using Geneious software (11.0.5) to remove ambiguously aligned terminal regions. The resulting alignments were concatenated and used to build a tree using maximum-likelihood approximation (Price et al., 2010), which was visualized using Dendroscope (v. 3.5.10). All reconstructed genomes were submitted to NCBI and can be accessed under the BioProject PRJNA488537 (accession numbers for the genomes can be found in Supplementary Table 9).

### Similarity Clustering of Genomes

High quality genomes were clustered in regard to their similarity using the dRep cluster module (Olm et al., 2017) with default parameters. As genomes were already quality checked, no CheckM (Parks et al., 2015) pre-filtering was performed. Circoletto (cutoff e-1^–50^) was used to visualize sequence similarity between the strains (Darzentas, 2010).

### Presence/Absence Calling of Microbial Genomes in Samples

To determine a precise presence/absence of each binned genome across the samples, we calculated the breadth via read mapping, i.e., the percentage of the genome that was covered with sequencing reads. Reads of each sample were cross-mapped against all recovered genomes using Bowtie2 2.2.6 in -sensitive mode (Langmead and Salzberg, 2012) and filtered for a maximum of five mismatches (2% error rate in 250 bp reads). The coverage of each nucleotide position for each scaffold and in each sample was determined as performed in the software uBin (Bornemann et al., 2020). After truncation of nucleotide positions by 250 bp at the ends of respective scaffolds to account for uneven mapping to the ends compared to the main body of the scaffolds, the breadth was calculated via the percentage of nucleotide positions with ≥3× coverage compared to the total (truncated) length of the respective genome.

### Analysis of Ribosomal Protein S3 to Measure Diversity

Scaffolds carrying an annotated ribosomal protein S3 gene (*rpS3*) were extracted and the coverage of the respective scaffolds was used to calculate a rank abundance curve. To assign precise taxonomies to the scaffolds making up the rank abundance curve, these were searched against the *rpS3* database of Hug and collaborators (Hug et al., 2016) using usearch (Edgar, 2010). Based on the percent identity of the *rpS3* gene with its best match in the database (cutoff 1 × 10^–5^), scaffolds were classified at species level (≥99%), genus level (88–98%), phylum level (60–87%), and domain level (<60%). The relative abundance of organisms was determined based on their proportion of the total abundance of all *rpS3*-carrying scaffolds. To differentiate unique and shared community members between the triplicate samples, scaffolds carrying *rps3* were clustered at 99% sequence identity using cd-hit (v4.6) (Li and Godzik, 2006). The taxonomic position of the *rpS3* sequences was determined by reconstructing a phylogeny placing them within the respective single-gene dataset from Hug et al. (2016). First, the *rpS3* sequences were aligned with MAFFT linsi v7.453 (Katoh and Standley, 2013) and for poorly aligning sequences we performed homology searches with BLASTp against NCBI’s nr (Altschul et al., 1990) to confirm their origin (e.g., eukaryotic, misannotations, potential misassemblies) and remove them. The remaining sequences were fused with the Hug dataset, realigned, and trimmed with ClipKIT (mode: kpic-gappy) (Steenwyk et al., 2020). A Maximum-Likelihood phylogeny was reconstructed in IQ-TREE 2 (Minh et al., 2020), under a model selected with ModelFinder (Kalyaanamoorthy et al., 2017), and branch supports calculated with 1000 ultrafast bootstraps (Hoang et al., 2018), 1000 SH-aLRT replicates (Guindon et al., 2010), and aBayes (Anisimova et al., 2011). The resulting phylogeny was visualized using Dendroscope (v. 3.5.10) (Huson and Scornavacca, 2012) and iTOL (Letunic and Bork, 2007).

### Metabolic Potential of Saccharibacteria

Genomes were annotated using the Genoscope platform MAGE (Vallenet et al., 2006, 2009). Analyses of the metabolic potential of the strains were performed by combining automated annotation from MAGE and manual curation using information from MetaCyc (Caspi et al., 2014), KEGG (Kanehisa and Goto, 2000) and UniProt (Bateman, 2019). Further analyses of protein sequences obtained from the genomes were done using PsortB (Yu et al., 2010), TMHMM (Krogh et al., 2001), and BLASTp (Altschul et al., 1990).

### Annotation of Potential Pathways for Hydrocarbon Degradation

Prodigal-predicted (Hyatt et al., 2010) proteins of reconstructed genomes were annotated via searching against UniPROT100 reference database (Bateman, 2019) using diamond (Buchfink et al., 2014). Annotations of hydrocarbon degrading genes were identified via word searches based on pathways for hydrocarbon degradation found in MetaCyc (Caspi et al., 2014). These genes were compared to the best blast hit obtained in the UniPROT100 predictions, and these candidate genes were further filtered based on a specific bitscore provided in Supplementary Table 8.

## Results

### Three Diesel-Amended Groundwater Microcosms Enrich for Bacteria Covering Four Different Phyla

**FIGURE 1:**
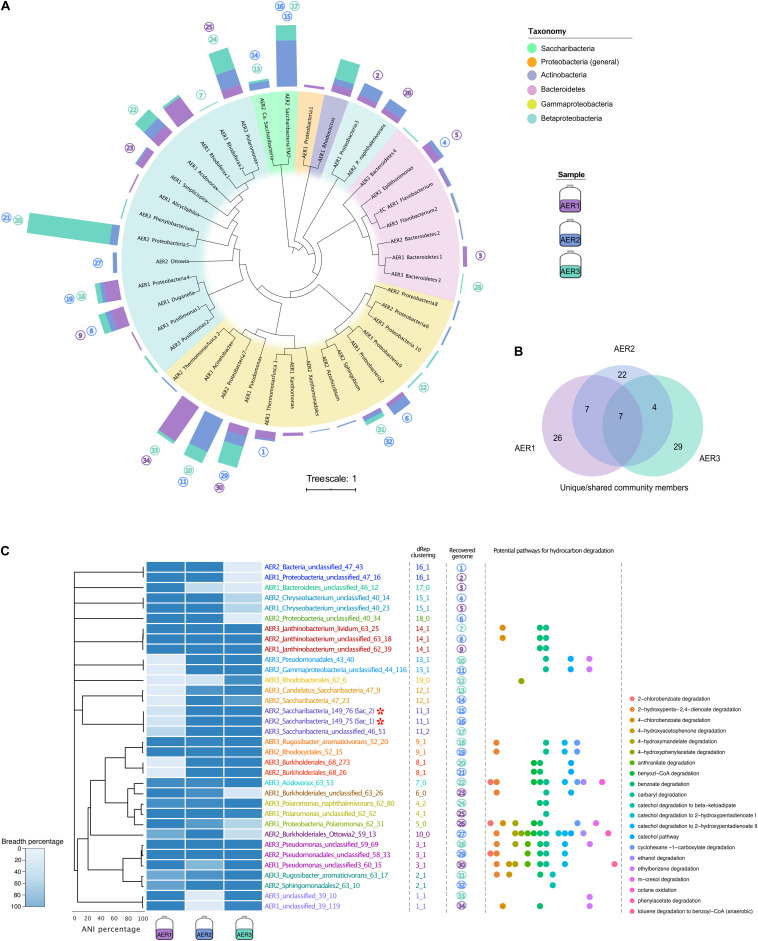
Community profile of diesel-amended enrichment cultures AER1, AER2, and AER3. **(A)** Diversity of the microbial community in the enrichments based on representative rpS3 sequences of each cluster. Bar charts show the abundance from normalized coverage of the scaffolds carrying the *rpS3* gene. The different bar colors correspond to which sample (AER1, AER2, or AER3) the respective organism was detected in. Circled numbers correlate to recovered genomes shown in panel **(C)**. Full tree of rpS3 sequences is provided as Supplementary File 1. **(B)** Venn-Diagram shows the unique and shared community profile amongst the three samples. **(C)** Breadth of coverage for the de-replicated set of recovered draft genomes, higher breadth values translate to a greater recovery of the respective draft genome in each sample, with Sac_1 and Sac_2 highlighted with red asterisks. The dendrogram is based on ANI values obtained with dRep. Output of the clustering of the recovered genomes with dRep is shown in the middle columns. First number in the clustering column is the output of MASH clustering. If genomes are found to be closely related in MASH, further ANI clustering is performed, denoted in the number after the underscore. Zero in this second number means no ANI clustering was performed. The different colored names denote the different clusters obtained. Circled numbers denote the recovered genomes. Please note that the breadth of genomes that were grouped by dRep into the same cluster was also highly similar, indicating that dRep and read-mapping based analyses agree well for species delineation. Potential for hydrocarbon degradation of the recovered genomes is shown, each colored circle denotes a different pathway, more information about it can be found in Supplementary Table 8.

Using ribosomal protein S3 (*rpS3*) as a marker, we identified 40 different organisms across four phyla in metagenomes of the three enrichment cultures (Figure 1A). The most diverse phylum was Proteobacteria with three individual lineages, while the most abundant phylum varied between enrichments cultures with Saccharibacteria, Proteobacteria and Bacteroidetes being the dominant taxa. Common representatives amongst the three samples belonged to Gammaproteobacteria and Betaproteobacteria. Actinobacteria and Bacteroidetes were also present with one and seven members, respectively. We reconstructed a total of 34 draft genomes with at least 70% completeness and less than 10% contamination (Supplementary Table 1). These draft genomes spanned 22 out of the 40 detected species based on *rpS3* sequence analysis and were grouped into 19 different genome clusters based on dRep. Interestingly, two members belonging to Saccharibacteria were detected in the respective rank abundance curves for both AER2 and AER3, and draft genomes could be obtained for these four Saccharibacteria. Previous analyses of the microbial community of AER2 showed that the enrichment was dominated by members of Gammaproteobacteria and *Rhodococcus*, pointing that these bacteria were the main key players for hydrocarbon degradation (Révész et al., 2020b).

### Diesel-Amended Groundwater Enrichments Show a High Variability in Organism Abundance

Based on *rpS3* genes, which we used as a proxy to determine the presence and absence of community members and to ascertain the replicability of the experiment, we found that the three enrichment cultures showed specific similarities and differences in their microbial community (Figure 1 and Supplementary Figures 1, 2). While the most abundant community members of AER2 and AER3 were below detection limit in AER1, the majority (52.5%) of organisms with a coverage cutoff greater than 4.1 were found in at least two of the enrichment cultures. Low abundant community members, which comprised almost half of the detected diversity (19 species, 47.5%), were not only seldomly binned but were also mostly unique to the specific enrichment cultures. At this stage, we speculate that the low abundance members were rather diverse due to the stochastic selection of DNA molecules for sequencing from the metagenomic libraries. Importantly, the most dominant member of each enrichment differed across all three replicates, although these were treated equally and also sequenced after the same incubation time. The Venn-Diagram in Figure 1 depicts the shared community members between the three enrichment cultures, leaving a core microbiome of seven members accountable for 17.5% of the overall community. Highly abundant community members showed greater success for genome reconstruction (75%) compared to low community members (12.5%; Supplementary Figure 1), which supports our conclusion of the stochastic selection of sequencing molecules to be attributable for the differences in the rare community.

Since the reconstruction of the community differences based on *rpS3* sequences only takes one gene into account, we investigated differences between the reconstructed genomes from each sample based on read mapping (allowing five mismatches per 250-bps read, which equals 2% difference) and calculation of the recovered genome fractions (breadth). dRep clustering assigned the genomes to 19 individual species. The results provided evidence that the recovered genomes showed great similarity between the enrichment cultures. Although the abundance of the genomes changed drastically across samples (Figure 1), the recovered communities showed great similarity with 11 out of 22 species being recovered at least twice.

### Dominance of Two Saccharibacteria Strains in Enrichment AER2

**FIGURE 2:**
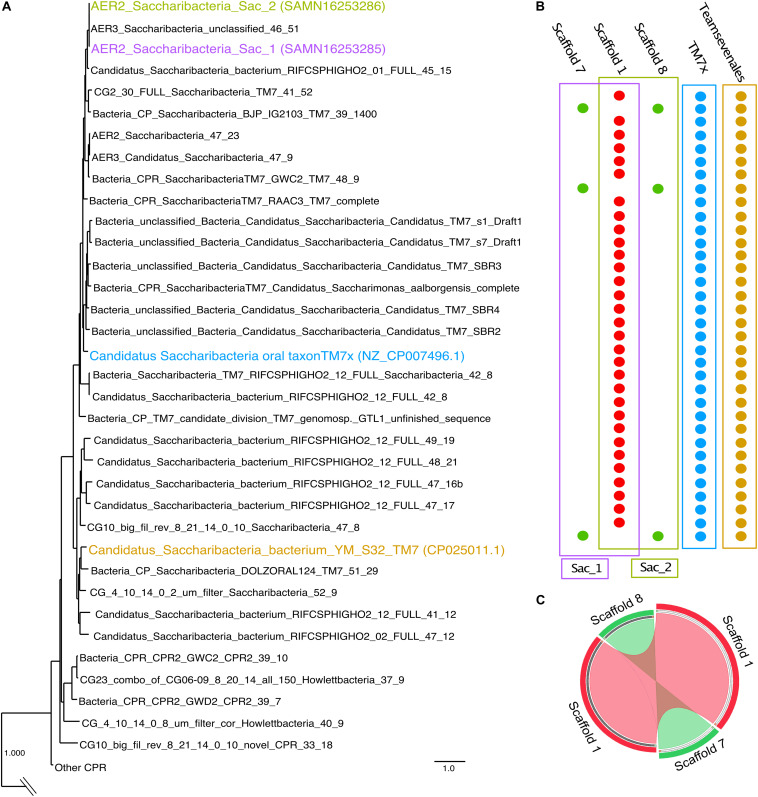
Phylogenomic tree of Saccharibacteria and their genome completeness. **(A)** Phylogenetic placement of Sac_1 and Sac_2 based on concatenation of 16 ribosomal proteins, highlighting Sac_1 and Sac_2, as well as TM7× and Teamsevenales, the cultivated representative with a circular complete genome and the strain identified by Starr et al. (2018), respectively. Tree was pruned from a full tree including all three domains of life which is available as Supplementary File 2. **(B)** Completeness of the Saccharibacteria genomes Sac_1 and Sac_2 based on presence/absence of SCGs, with TM7× and Teamsevenales for comparison. Scaffolds 7 and 8, that make up the genomes of Sac_1 and Sac_2, respectively, are shown to compensate for absence of SCGs in Scaffold 1. Each bullet represents a different SCG per line, and each colored outline groups the SCGs found in each strain. The complete list of SCGs is provided as Supplementary Table 7. **(C)** Circos plot comparing the two Saccharibacteria genomes of Sac_1 and Sac_2. While Scaffold 1 is identical, Scaffold 8 and 7 also show high similarity based on blastn (cutoff 1e^–50^).

Based on *rpS3* sequence analysis, sample AER2 was dominated by a member of the Saccharibacteria, for which we also recovered a genome bin. The bin had three scaffolds totaling 1.3 Mbps and an average coverage of 118. Interestingly, the coverage of the bin varied greatly between scaffolds, from 149.7 to 75.4 (and 76.0, respectively, for the three scaffolds). The percent contamination based on CheckM was 11% and the strain heterogeneity 92.9%. Based on scaffold alignments, the two low abundant scaffolds in the megabin aligned with each other very well, had redundant SCGs, and their summed coverage of 149.7 also complemented the bigger scaffold. Based on the alignment, the redundancy in SCGs, and the nearly double coverage of the larger scaffold, we concluded that the two short scaffolds were separately assembled due to strain variation (Figure 2B). Consequently, we were able to bin two separate Saccharibacteria genomes (1.06 Mbps each) from this megabin, which resulted each in 92% completeness and 0% contamination and strain heterogeneity. Based on the present of bacterial SCGs and comparing them to those in TM7× and Teamsevenales, the genomes were estimated to be essentially complete (<100%; Figure 2B; Supplementary Table 7), since it is known that CPR bacteria lack, e.g., certain ribosomal proteins (Brown et al., 2015). It should be noted that we do not report genome completeness of 100% due to the lack of a closed circular genome for the Saccharibacteria strains. Strain AER2_Sac1 is made up of the large scaffold (Scaffold 1) and the small scaffold with a coverage of 75.4 (Scaffold 7), while strain AER2_Sac2 is composed of the large scaffold but with the small scaffold of coverage 76.0 (Scaffold 8) (Figure 2B). We conclude that the separate assembly of the two Saccharibacteria strains underpins strain heterogeneity arising from these genomes. The genomic region that differed between the two strains encoded for ten different proteins. For seven out of these ten proteins we were not able to determine a potential function based on protein annotation. The remaining three proteins were annotated as Clp protease, a hydro-lyase, and a 50S ribosomal subunit protein L34.

### Saccharibacteria Have Limited Metabolic Capacity Centered Around Exogenous Carbon and Energy Sources

Since the two dominant strains AER2_Sac1 and AER2_Sac2 were nearly identical based on the assembled genomes, we analyzed their metabolic potential in tandem. Similar to previously published Saccharibacteria genomes, pathways for *de novo* biosynthesis of fatty acids, nucleotides, and amino acids were incomplete (see Supplementary Tables 2, 3), although ten out of 14 enzymes for pyrimidine biosynthesis were identified. For purine biosynthesis, the genomes encoded 6 out of 12 reported enzymes necessary for *de novo* synthesis. Since the genomes were essentially complete (only in two scaffolds and all SCG of Saccharibacteria present), we conclude that the Saccharibacteria were unable to synthesize their own nucleotides. In fact, we identified 14 different nucleases encoded in each the two Saccaribacteria genomes, highlighting their potential to break down (external) DNA for retrieval of nucleotides. The production of S-adenosyl-methionine from methionine was also encoded in the genome likely providing necessary C_1_-groups or is involved in amino acid fermentation.

Overall the dominant Saccharibacteria strains seemed to obtain most of their organic carbon and molecular building blocks from the environment, e.g., from other organisms. We identified proteases, aminopeptidases, carboxypeptidases, and metalloproteases along with several transporters for amino acids and oligopeptides to compensate for the lack of amino acid biosynthesis (see Supplementary Tables 4, 5). Specifically, a glutamate/Na^+^ symporter was annotated, which provides a carbon and nitrogen source to the Saccharibacteria. Conversion of simple amino acids like cysteine from alanine (sulfur source), and glycine from serine were also present. tRNA charging ligases were also found, with only asparagine and glutamine missing. In combination with ribosomal proteins, we consequently identified a near complete set of genes for *de novo* protein biosynthesis.

**FIGURE 3:**
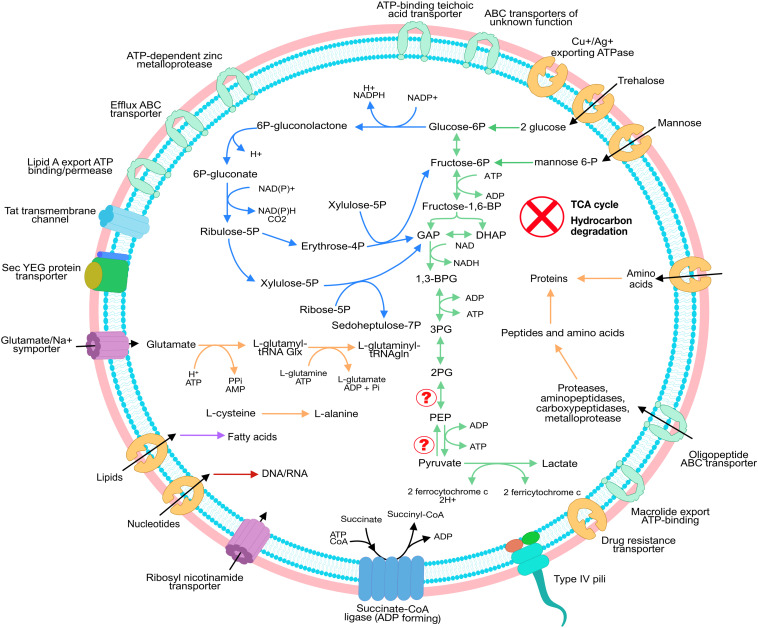
Metabolic reconstruction of Sac_1 And Sac_2. The Saccharibacteria strains show minimal metabolic capacity. No TCA cycle was detected but the strains encode for an almost complete glycolysis as well as a complete PPP. Please note that we detected several transporters with unknown function, which might aid in the uptake of, e.g., nucleotides. Supplementary Tables 2–5 give the complete lists of the annotated pathways and transporters found for Sac_1 and Sac_2.

While 23 out of the 34 reconstructed genomes encoded for at least one hydrocarbon degradation pathway (Figure 1C and Supplementary Table 8), the potential for hydrocarbon degradation was absent in all Saccharibacteria genomes. In detail, analyses for the presence of enzymes related to hydrocarbon degradation in the Saccharibacteria genomes, e.g., for degradation of BTEX or aromatic compounds, did not yield any hits with identities above 38% (e.g., we searched for 4-formylbenzenesulfonate dehydrogenase (tsaC1) and the Toluene-4-monooxygenase system, ferredoxin–NAD(+) reductase component (tmoF)). Instead, we identified a near complete pathway for sugar fermentation. Transporters for mannose and trehalose were encoded in the Saccharibacteria genome aligning with the ability to convert mannose and trehalose to glucose-6P and fructose-6P, respectively. These products could then be funneled into a near-complete glycolysis (Figure 3). The missing enzymatic steps in glycolysis are likely compensated by a near complete pentose phosphate pathway (PPP). For instance, the PPP can compensate for the 6-phosphofructokinase to obtain glyceraldehyde 3-phosphate (GAP) and subsequently pyruvate. The absence of a TCA cycle (except for the ADP-forming succinate-CoA ligase), and the presence of lactate dehydrogenases suggests the fermentation of the pyruvate to lactate and the re-oxidation of the reduction equivalents NAD^+^. These reduction equivalents are either taken up from the environment via respective transporters encoded in the Saccharibacteria genome or are synthesized via a salvage pathway compensating for the missing NAD biosynthesis.

## Discussion

The current body of literature suggests an involvement of Saccharibacteria in hydrocarbon degradation, however, the metabolic potential of these organisms from hydrocarbon contaminated sites or hydrocarbon-amended enrichments has not been elucidated so far. To fill this knowledge gap, we reconstructed near-complete genomes of highly abundant Saccharibacteria from diesel-amended enrichment cultures and performed metabolic analyses to study the potential of these organisms in hydrocarbon degradation and other carbon related cellular processes.

Analyses of the genomic information of Saccharibacteria Sac_1 and Sac_2, and CPR in general, demonstrate that their metabolic potential is very limited, lacking the capacity for *de novo* synthesis of important molecular building blocks (Brown et al., 2015; Jaffe et al., 2020). Similar to some previously reported members of the CPR, Sac_1 and Sac_2 have the capacity to synthesize peptidoglycan and likely have a fermentation-based lifestyle (Castelle et al., 2018; Jaffe et al., 2020). Although previously reported glycolysis and PPP in CPR were frequently incomplete, the Saccharibacteria genomes recovered in this study can make use of various hexoses and break them down to lactate through glycolysis and the PPP. Nevertheless, Sac_1 and Sac_2 had a limited metabolism with heavily incomplete lipid and nucleotide biosynthesis pathways and thus likely performed a symbiotic lifestyle. To overcome limitations of missing biosynthetic pathways of, e.g., amino acids and vitamins, Saccharibacteria can make use of their various transporters as previously reported (Castelle et al., 2018). For instance, CPR genomes can contain up to 75 transporters per genome, although most are of unknown function (Castelle et al., 2018). Genes for the synthesis of type-IV pili are fairly widely distributed within CPR (Castelle et al., 2018; McLean et al., 2018), and these systems are reported to be involved in the uptake of DNA (Averhoff and Friedrich, 2003) and were also present in Sac_1 and Sac_2. The two genomes also encoded for several transporters for retrieval of peptides for degradation to amino acids or directly for the uptake of external amino acids, such as glutamate. The lack of enzymes of both Sac_1 and Sac_2 related to hydrocarbon degradation and their ability to uptake sugars and other molecules from the environment, suggests that these strains are not directly involved in the degradation of petroleum compounds, but rather make use of the molecules and byproducts of the other bacteria.

Members of the phylum Saccharibacteria have been identified in petroleum-contaminated sites (Liu et al., 2020; Révész et al., 2020b), some of which reported a potential role of these bacteria in the degradation of hydrocarbons (Luo et al., 2009; Xie et al., 2011). Analyses of enrichment cultures set up to study the uptake of labeled ^13^C_6_ toluene and ^13^C_6_ benzene showed that the 16S rRNA gene fragments obtained from T-RFLP belonged to members of the Saccharibacteria. In the case of the toluene enrichment cultures, only that particular T-RFLP fragment from the community was seen to increase over three time points during incubation (Luo et al., 2009). On the other hand, ^13^C-labeling of DNA from benzene was not only identified for Saccharibacteria but also in other bacteria and the respective authors concluded that these organisms metabolized benzene (Xie et al., 2011). In comparison, Starr and co-workers studied the carbon flux from plants (*Avena fatua*) to rhizosphere microorganisms by using ^13^C-labeled CO_2_ and SIP analyses of the community DNA from the surrounding soil. They identified a member of Saccharibacteria in the heavy fraction, meaning it had incorporated ^13^C into its DNA, and they were able to obtain its complete genome. Analyses of this genome demonstrated that the Saccharibacteria were unable to *de novo* produce the nucleotides for DNA and RNA synthesis, and thus depended on the uptake of externally derived nucleotides. The authors inferred that the Saccharibacteria partakes in cross-feeding, where it uptakes the labeled nucleotides from other bacteria in the community (most likely its host), which in turn have produced these precursors using plant-derived labeled carbon (Starr et al., 2018). Consequently, ^13^C labeling of the DNA of organisms that derive their nucleotides from external sources does not allow the conclusion that they metabolized the ^13^C-labeled substrate themselves. If we extrapolate these findings to the toluene and benzene experiments that identified labeled Saccharibacteria DNA (Luo et al., 2009; Xie et al., 2011), it would necessarily mean that the labeled Saccharibacteria never metabolized the respective substrate either. In this scenario, hydrocarbon degraders were responsible for the direct uptake and breakdown of the labeled toluene and benzene, and the Saccharibacteria would make use of the labeled nucleotides, and other molecular precursors, produced by the hydrocarbon degraders. Indeed, based on 16S rRNA gene analysis the genome that we recovered from our analysis belonged to the same genus of Saccharibacteria as one of the organisms identified by Luo and co-workers (Supplementary File 3; Yarza et al., 2014). This combined with the fact that all so far identified CPR bacteria are devoid of complete nucleotide synthesis pathways, renders the above-mentioned scenario extremely likely. Some studies have reported low abundance of hydrocarbon degraders in the microbial community, hinting that cross-feeding would play an important role in maintaining the community stable. This due to some members being directly responsible for hydrocarbon degradation, producing by-products that other members could make use of, or even some bacteria could feed on necromass (Taubert et al., 2012; Melkonian et al., 2020). Interestingly, the toluene and benzene amended enrichment cultures did not show another organism apart from Saccharibacteria with ^13^C-labeled DNA. This suggests that the detected Saccharibacteria strains had a parasitic behavior toward their respective host, i.e., scavenging the nucleotides once the host cell has been compromised and lysed. This parasitic behavior has indeed been very well documented for other Saccharibacteria like TM7× (He et al., 2015; McLean et al., 2016). This is further evidenced by the finding that ^13^C-labeled *Polaromonas* sp., *Sphingomonadaceae* and *Acidobacterium* were detected in Saccharibacteria-free enrichments of the same study, and these bacteria could serve as potential hosts for Saccharibacteria (Xie et al., 2011). We were also able to detect members belonging to *Polaromonas* sp. and *Sphingobium* sp. in the three enrichment samples, but our results do not allow any conclusions if these or other microbes act as potential hosts for the Saccharibacteria strains. We closed the knowledge gap regarding the role of Saccharibacteria in hydrocarbon-enriched environments by providing evidence that near-complete Saccharibacteria genomes dominant in hydrocarbon-amended enrichments do not possess the capacity for hydrocarbon breakdown and rather act as sinks of organic carbon.

## Conclusion

In this study, we compared the reproducibility of hydrocarbon-amended microcosms from the same ecosystem. Our results demonstrated a high recovery rate of same species and strains that are highly abundant but low similarity between abundance patterns. Based on the reconstruction of Saccharibacteria genomes from these enrichment cultures we challenged the results of previous studies that suggested an involvement of Saccharibacteria in hydrocarbon degradation. We performed a thorough metabolic analysis of two abundant Saccharibacteria strains, which revealed no evidence for hydrocarbon degradation by these organisms. Instead, we demonstrate that these Saccharibacteria are similar in their metabolism to previously recovered organisms from this phylum with a limited metabolism centered around peptide and sugar degradation. We conclude that Saccharibacteria live off molecular building blocks from other organisms, likely hydrocarbon degraders, and consequently represent a sink of organic carbon in hydrocarbon-fueled environments.

## Data Availability Statement

All reconstructed genomes were submitted to NCBI and can be accessed under the BioProject PRJNA488537 (accession numbers for the genomes can be found in Supplementary Table 9).

## Author Contributions

PF-G performed the genome-resolved metagenomics, community analyses, and phylogenomics. PF-G, TB, and AP performed the bioinformatics. PA performed the phylogenetics. JP performed analyses of the presence of hydrocarbon degradation pathways. FR executed the enrichment cultures. PF-G and CvH performed the metabolic analyses. AT designed the study. PF-G and AP wrote the manuscript with revisions from all co-authors. All authors contributed to the article and approved the submitted version.

## Conflict of Interest

The authors declare that the research was conducted in the absence of any commercial or financial relationships that could be construed as a potential conflict of interest.

## Footnotes

1 sourceforge.net/projects/bbmap/

## References

[B1] Abdel-Megeed A. Al-Harbi N. Al-Deyab S. (2010). Hexadecane degradation by bacterial strains isolated from contaminated soils. *Afr. J. Biotechnol.* 9 7487–7494. 10.5897/ajb10.638

[B2] Altschul S. F. Gish W. Miller W. Myers E. W. Lipman D. J. (1990). Basic local alignment search tool. *J. Mol. Biol.* 215 403–410. 10.1016/S0022-2836(05)80360-2 2231712

[B3] Anisimova M. Gil M. Dufayard J. F. Dessimoz C. Gascuel O. (2011). Survey of branch support methods demonstrates accuracy, power, and robustness of fast likelihood-based approximation schemes. *Syst. Biol.* 60 685–699. 10.1093/sysbio/syr041 21540409 PMC3158332

[B4] Anjum N. A. Gill S. S. Tuteja N. (2017). *Enhancing Cleanup of Environmental Pollutants*, Vol. 1 New York, NY: Springer International Publishing, 1–327. 10.1007/978-3-319-55426-6

[B5] Averhoff B. Friedrich A. (2003). Type IV pili-related natural transformation systems: DNA transport in mesophilic and thermophilic bacteria. *Arch. Microbiol.* 180 385–393. 10.1007/s00203-003-0616-6 14593449

[B6] Bateman A. (2019). UniProt: a worldwide hub of protein knowledge. *Nucleic Acids Res.* 47 D506–D515. 10.1093/nar/gky1049 30395287 PMC6323992

[B7] Benedek T. Táncsics A. Szabó I. Farkas M. Szoboszlay S. Fábián K. (2016). Polyphasic analysis of an Azoarcus-Leptothrix-dominated bacterial biofilm developed on stainless steel surface in a gasoline-contaminated hypoxic groundwater. *Environ. Sci. Pollut. Res.* 23 9019–9035. 10.1007/s11356-016-6128-0 26825521

[B8] Berthe-Corti L. Fetzner S. (2002). Bacterial metabolism of n-alkanes and ammonia under oxic, suboxic and anoxic conditions. *Acta Biotechnol.* 22 299–336. 10.1002/1521-3846(200207)22:3/4<299::AID-ABIO299<3.0.CO;2-F

[B9] Bor B. Collins A. J. Murugkar P. P. Balasubramanian S. To T. T. Hendrickson E. L. (2020). Insights obtained by culturing saccharibacteria with their bacterial hosts. *J. Dental Res.* 99 685–694. 10.1177/0022034520905792 PMC7243422 32075512

[B10] Bor B. McLean J. S. Foster K. R. Cen L. To T. T. Serrato-Guillen A. (2018). Rapid evolution of decreased host susceptibility drives a stable relationship between ultrasmall parasite TM7x and its bacterial host. *Proc. Natl. Acad. Sci. U.S.A.* 115 12277–12282. 10.1073/pnas.1810625115 30442671 PMC6275545

[B11] Bornemann T. L. Esser S. P. Stach T. L. Burg T. Probst A. J. (2020). uBin-a manual refining tool for metagenomic bins designed for educational purposes. *bioRxiv* [Preprint]. 10.1017/CBO9781107415324.004

[B12] Brown C. T. Hug L. A. Thomas B. C. Sharon I. Castelle C. J. Singh A. (2015). Unusual biology across a group comprising more than 15% of domain Bacteria. *Nature* 523 208–211. 10.1038/nature14486 26083755

[B13] Buchfink B. Xie C. Huson D. H. (2014). Fast and sensitive protein alignment using DIAMOND. *Nat. Methods* 12 59–60. 10.1038/nmeth.3176 25402007

[B14] Caspi R. Altman T. Billington R. Dreher K. Foerster H. Fulcher C. A. (2014). The MetaCyc database of metabolic pathways and enzymes and the BioCyc collection of Pathway/Genome Databases. *Nucleic Acids Res.* 42 459–471. 10.1093/nar/gkt1103 24225315 PMC3964957

[B15] Castelle C. J. Brown C. T. Anantharaman K. Probst A. J. Huang R. H. Banfield J. F. (2018). Biosynthetic capacity, metabolic variety and unusual biology in the CPR and DPANN radiations. *Nat. Rev. Microbiol.* 16 629–645. 10.1038/s41579-018-0076-2 30181663

[B16] Chaillan F. Le Flèche A. Bury E. Phantavong Y.-H. Grimont P. Saliot A. (2004). Identification and biodegradation potential of tropical aerobic hydrocarbon-degrading microorganisms. *Res. Microbiol.* 155 587–595. 10.1016/j.resmic.2004.04.006 15313261

[B17] Chong C. W. Silvaraj S. Supramaniam Y. Snape I. Tan I. K. P. (2018). Effect of temperature on bacterial community in petroleum hydrocarbon-contaminated and uncontaminated Antarctic soil. *Polar Biol.* 41 1763–1775. 10.1007/s00300-018-2316-3

[B18] Cross K. L. Campbell J. H. Balachandran M. Campbell A. G. Cooper S. J. Griffen A. (2019). Targeted isolation and cultivation of uncultivated bacteria by reverse genomics. *Nat. Biotechnol.* 37 1314–1321. 10.1038/s41587-019-0260-6 31570900 PMC6858544

[B19] Darzentas N. (2010). Circoletto: visualizing sequence similarity with Circos. *Bioinformatics* 26 2620–2621. 10.1093/bioinformatics/btq484 20736339

[B20] Dick G. J. Andersson A. F. Baker B. J. Simmons S. L. Thomas B. C. Yelton A. P. (2009). Community-wide analysis of microbial genome sequence signatures. *Genome Biol.* 10 R85. 10.1186/gb-2009-10-8-r85 19698104 PMC2745766

[B21] Edgar R. C. (2004). MUSCLE: multiple sequence alignment with high accuracy and high throughput. *Nucleic Acids Res.* 32 1792–1797. 10.1093/nar/gkh340 15034147 PMC390337

[B22] Edgar R. C. (2010). Search and clustering orders of magnitude faster than BLAST. *Bioinformatics* 26 2460–2461. 10.1093/bioinformatics/btq461 20709691

[B23] Farhadian M. Vachelard C. Duchez D. Larroche C. (2008). In situ bioremediation of monoaromatic pollutants in groundwater: a review. *Bioresour. Technol.* 99 5296–5308. 10.1016/j.biortech.2007.10.025 18054222

[B24] Foster S. S. D. Chilton P. J. (2003). Groundwater: the processes and global significance of aquifer degradation. *Philos. Trans. R. Soc. B Biol. Sci.* 358 1957–1972. 10.1098/rstb.2003.1380 14728791 PMC1693287

[B25] Guindon S. Dufayard J. F. Lefort V. Anisimova M. Hordijk W. Gascuel O. (2010). New algorithms and methods to estimate maximum-likelihood phylogenies: assessing the performance of PhyML 3.0. *Syst. Biol.* 59 307–321. 10.1093/sysbio/syq010 20525638

[B26] He X. McLean J. S. Edlund A. Yooseph S. Hall A. P. Liu S. Y. (2015). Cultivation of a human-associated TM7 phylotype reveals a reduced genome and epibiotic parasitic lifestyle. *Proc. Natl. Acad. Sci. U.S.A.* 112 244–249. 10.1073/pnas.1419038112 25535390 PMC4291631

[B27] Hoang D. T. Chernomor O. von Haeseler A. Minh B. Q. Vinh L. S. (2018). UFBoot2: improving the ultrafast bootstrap approximation. *Mol. Biol. Evol.* 35 518–522. 10.5281/zenodo.854445 29077904 PMC5850222

[B28] Hug L. A. Baker B. J. Anantharaman K. Brown C. T. Probst A. J. Castelle C. J. (2016). A new view of the tree of life. *Nat. Microbiol.* 1 1–6. 10.1038/nmicrobiol.2016.48 27572647

[B29] Huson D. H. Scornavacca C. (2012). Dendroscope 3: an interactive tool for rooted phylogenetic trees and networks. *Syst. Biol.* 61 1061–1067. 10.1093/sysbio/sys062 22780991

[B30] Hyatt D. Chen G.-L. LoCascio P. F. Land M. L. Larimer F. W. Hauser L. J. (2010). Prodigal: prokaryotic gene recognition and translation initiation site identification. *BMC Bioinformatics* 11:119. 10.1186/1471-2105-11-119 20211023 PMC2848648

[B31] Jaffe A. L. Castelle C. J. Matheus Carnevali P. B. Gribaldo S. Banfield J. F. (2020). The rise of diversity in metabolic platforms across the Candidate Phyla radiation. *BMC Biol.* 18:69. 10.1186/s12915-020-00804-5 32560683 PMC7304191

[B32] Joshi N. A. Fass J. N. (2011). *Sickle**: A Sliding-window, Adaptive, Quality-based trimming Tool for FastQ Files* ((Version 1.33)). Available online at: https://github.com/najoshi/sickle (accessed July 22, 2020).

[B33] Kalyaanamoorthy S. Minh B. Q. Wong T. K. F. Von Haeseler A. Jermiin L. S. (2017). ModelFinder: fast model selection for accurate phylogenetic estimates. *Nat. Methods* 14 587–589. 10.1038/nmeth.4285 28481363 PMC5453245

[B34] Kanehisa M. Goto S. (2000). KEGG: kyoto encyclopedia of genes and genomes. *Nucleic Acids Res.* 28 27–30. 10.3892/ol.2020.11439 10592173 PMC102409

[B35] Katoh K. Standley D. M. (2013). MAFFT multiple sequence alignment software version 7: improvements in performance and usability. *Mol. Biol. Evol.* 30 772–780. 10.1093/molbev/mst010 23329690 PMC3603318

[B36] Krogh A. Larsson B. Von Heijne G. Sonnhammer E. L. L. (2001). Predicting transmembrane protein topology with a hidden Markov model: application to complete genomes. *J. Mol. Biol.* 305 567–580. 10.1006/jmbi.2000.4315 11152613

[B37] Langmead B. Salzberg S. L. (2012). Fast gapped-read alignment with Bowtie 2. *Nat. Methods* 9 357–359. 10.1038/nmeth.1923 22388286 PMC3322381

[B38] Letunic I. Bork P. (2007). Interactive Tree Of Life (iTOL): an online tool for phylogenetic tree display and annotation. *Bioinformatics* 23 127–128. 10.1093/bioinformatics/btl529 17050570

[B39] Li W. Godzik A. (2006). Cd-hit: a fast program for clustering and comparing large sets of protein or nucleotide sequences. *Bioinformatics* 22 1658–1659. 10.1093/bioinformatics/btl158 16731699

[B40] Liu H. Gao H. Wu M. Ma C. Wu J. Ye X. (2020). Distribution characteristics of bacterial communities and hydrocarbon degradation dynamics during the remediation of petroleum-contaminated soil by enhancing moisture content. *Microbial Ecol.* 80 202–211. 10.1007/s00248-019-01476-7 31955225

[B41] Luo C. Xie S. Sun W. Li X. Cupples A. M. (2009). Identification of a novel toluene-degrading bacterium from the candidate phylum TM7, as determined by DNA stable isotope probing. *Appl. Environ. Microbiol.* 75 4644–4647. 10.1128/AEM.00283-09 19447956 PMC2704817

[B42] Mahmoud G. A. E. Bagy M. M. K. (2018). “Microbial degradation of petroleum hydrocarbons,” in *Microbial Action on Hydrocarbons* eds Kumar V. Kumar M. Prasad R. (Singapore: Springer). 10.1007/978-981-13-1840-5_12

[B43] McLean J. S. Bor B. To T. T. Liu Q. Kerns K. A. Solden L. (2018). Independent acquisition and adaptation of ultra-small bacteria with reduced genomes from the phylum saccharibacteria to human hosts. *SSRN Electronic J.* 43. 10.2139/ssrn.3192029 32864128

[B44] McLean J. S. Liu Q. Bor B. Bedree J. K. Cen L. Watling M. (2016). Draft genome sequence of Actinomyces odontolyticus subsp. actinosynbacter strain XH001, the basibiont of an oral TM7 epibiont. *Genome Announcements* 4 e1685–e1615. 10.1128/genomeA.01685-15 26847892 PMC4742689

[B45] Melkonian C. Fillinger L. Atashgahi S. Nunes da Rocha U. Kuiper E. Olivier B. (2020). Biodiversity and niche partitioning in an anaerobic benzene degrading culture. *bioRxiv* [Preprint]. 10.1101/2020.07.17.208124

[B46] Minh B. Q. Schmidt H. A. Chernomor O. Schrempf D. Woodhams M. D. Von Haeseler A. (2020). IQ-TREE 2: new models and efficient methods for phylogenetic inference in the genomic Era. *Mol. Biol. Evol.* 37 1530–1534. 10.1093/molbev/msaa015 32011700 PMC7182206

[B47] Nurk S. Meleshko D. Korobeynikov A. Pevzner P. A. (2017). MetaSPAdes: a new versatile metagenomic assembler. *Genome Res.* 27 824–834. 10.1101/gr.213959.116 28298430 PMC5411777

[B48] Olm M. R. Brown C. T. Brooks B. Banfield J. F. (2017). DRep: a tool for fast and accurate genomic comparisons that enables improved genome recovery from metagenomes through de-replication. *ISME J.* 11 2864–2868. 10.1038/ismej.2017.126 28742071 PMC5702732

[B49] Parks D. H. Imelfort M. Skennerton C. T. Hugenholtz P. Tyson G. W. (2015). CheckM: assessing the quality of microbial genomes recovered from isolates, single cells, and metagenomes. *Genome Res.* 25 1043–1055. 10.1101/gr.186072.114 25977477 PMC4484387

[B50] Price M. N. Dehal P. S. Arkin A. P. (2010). FastTree 2 - Approximately maximum-likelihood trees for large alignments. *PLoS One* 5:e9490. 10.1371/journal.pone.0009490 20224823 PMC2835736

[B51] Prince R. C. Amande T. J. McGenity T. J. (2019). “Prokaryotic hydrocarbon degraders,” in *Taxonomy, Genomics and Ecophysiology of Hydrocarbon-Degrading Microbes. Handbook of Hydrocarbon and Lipid Microbiology*, ed. Timmis K. N. (Berlin: Springer), 1–39. 10.1007/978-3-540-77587-4_118

[B52] Probst A. J. Castelle C. J. Singh A. Brown C. T. Anantharaman K. Sharon I. (2017). Genomic resolution of a cold subsurface aquifer community provides metabolic insights for novel microbes adapted to high CO2 concentrations. *Environ. Microbiol.* 19 459–474. 10.1111/1462-2920.13362 27112493

[B53] Quast C. Pruesse E. Yilmaz P. Gerken J. Schweer T. Yarza P. (2013). The SILVA ribosomal RNA gene database project: improved data processing and web-based tools. *Nucleic Acids Res.* 41 590–596. 10.1093/nar/gks1219 23193283 PMC3531112

[B54] Révész F. Farkas M. Kriszt B. Szoboszlay S. Benedek T. Táncsics A. (2020a). Effect of oxygen limitation on the enrichment of bacteria degrading either benzene or toluene and the identification of Malikia spinosa (Comamonadaceae) as prominent aerobic benzene-, toluene-, and ethylbenzene-degrading bacterium: enrichment, isolation and whole genome analysis. *Environ. Sci. Pollut. Res.* 27 31130–31142. 10.1007/s11356-020-09277-z 32474783 PMC7392937

[B55] Révész F. Figueroa-Gonzalez P. A. Probst A. J. Kriszt B. Banerjee S. Szoboszlay S. (2020b). Microaerobic conditions caused the overwhelming dominance of *Acinetobacter* spp. and the marginalization of *Rhodococcus* spp. in diesel fuel/crude oil mixture-amended enrichment cultures. *Arch. Microbiol.* 202 329–342. 10.1007/s00203-019-01749-2 31664492 PMC7012980

[B56] Salam L. B. Ilori M. O. Amund O. O. LiiMien Y. Nojiri H. (2018). Characterization of bacterial community structure in a hydrocarbon-contaminated tropical African soil. *Environ. Technol.* 39 939–951. 10.1080/09593330.2017.1317838 28393681

[B57] Schwarzenbach R. P. Egli T. Hofstetter T. B. von Gunten U. Wehrli B. (2010). Global water pollution and human health. *Annu. Rev. Environ. Resour.* 35 109–136. 10.1146/annurev-environ-100809-125342

[B58] Singh S. N. Kumari B. Mishra S. (2012). *Microbial Degradation of Alkanes.* Berlin: Springer, 439–469. 10.1007/978-3-642-23789-8

[B59] Starr E. Shi S. Blazewicz S. Probst A. Herman D. Firestone M. (2018). Stable isotope informed genome-resolved metagenomics reveals that Saccharibacteria utilize microbially processed plant derived carbon. *Microbiome* 6:122 10.1101/211649 PMC6031116 29970182

[B60] Steenwyk J. L. Buida T. J. Li Y. Shen X.-X. Rokas A. (2020). ClipKIT: a multiple sequence alignment-trimming algorithm for accurate phylogenomic inference. *bioRxiv* [Preprint]. 10.1101/2020.06.08.140384 PMC7735675 33264284

[B61] Steiert J. G. Pignatello J. J. Crawford R. L. (1987). Degradation of chlorinated phenols by a pentachlorophenol-degrading bacterium. *Appl. Environ. Microbiol.* 53 907–910. 10.1128/aem.53.5.907-910.1987 3606097 PMC203784

[B62] Suzek B. E. Huang H. McGarvey P. Mazumder R. Wu C. H. (2007). UniRef: comprehensive and non-redundant UniProt reference clusters. *Bioinformatics* 23 1282–1288. 10.1093/bioinformatics/btm098 17379688

[B63] Taubert M. Vogt C. Wubet T. Kleinsteuber S. Tarkka M. T. Harms H. (2012). Protein-SIP enables time-resolved analysis of the carbon flux in a sulfate-reducing, benzene-degrading microbial consortium. *ISME J.* 6 2291–2301. 10.1038/ismej.2012.68 22791237 PMC3504967

[B64] Throne-Holst M. Wentzel A. Ellingsen T. E. Kotlar H. K. Zotchev S. B. (2007). Identification of novel genes involved in long-chain n-alkane degradation by *Acinetobacter* sp. strain DSM 17874. *Appl. Environ. Microbiol.* 73 3327–3332. 10.1128/AEM.00064-07 17400787 PMC1907095

[B65] Utter D. R. He X. Cavanaugh C. M. McLean J. S. Bor B. (2020). The saccharibacterium TM7x elicits differential responses across its host range. *ISME J.* 14 3054–3067. 10.1038/s41396-020-00736-6 32839546 PMC7784981

[B66] Vallenet D. Engelen S. Mornico D. Cruveiller S. Fleury L. Lajus A. (2009). MicroScope: a platform for microbial genome annotation and comparative genomics. *Database* 2009 1–12. 10.1093/database/bap021 20157493 PMC2790312

[B67] Vallenet D. Labarre L. Rouy Z. Barbe V. Bocs S. Cruveiller S. (2006). MaGe: a microbial genome annotation system supported by synteny results. *Nucleic Acids Res.* 34 53–65. 10.1093/nar/gkj406 16407324 PMC1326237

[B68] Xie S. Sun W. Luo C. Cupples A. M. (2011). Novel aerobic benzene degrading microorganisms identified in three soils by stable isotope probing. *Biodegradation* 22 71–81. 10.1007/s10532-010-9377-5 20549308

[B69] Yarza P. Yilmaz P. Pruesse E. Glöckner F. O. Ludwig W. Schleifer K. H. (2014). Uniting the classification of cultured and uncultured bacteria and archaea using 16S rRNA gene sequences. *Nat. Rev. Microbiol.* 12 635–645. 10.1038/nrmicro3330 25118885

[B70] Yu N. Y. Wagner J. R. Laird M. R. Melli G. Rey S. Lo R. (2010). PSORTb 3.0: improved protein subcellular localization prediction with refined localization subcategories and predictive capabilities for all prokaryotes. *Bioinformatics* 26 1608–1615. 10.1093/bioinformatics/btq249 20472543 PMC2887053

