# Supplementary material for: Saccharibacteria as Organic Carbon Sinks in Hydrocarbon-Fueled Communities
Source: Front Microbiol. 2020 Dec 23;11:587782. doi: 10.3389/fmicb.2020.587782 (PMC7786006; doi:10.3389/fmicb.2020.587782)

Supplementary information for:

**Saccharibacteria as organic carbon sinks in hydrocarbon-fueled communities**

Perla Abigail Figueroa-Gonzalez^1^, Till L.V. Bornemann^1^, Panagiotis S. Adam^1^, Julia Plewka^1^, Fruzsina Révész^2,3^, Christian A. von Hagen^1^, András Táncsics^2,3^*, Alexander J. Probst^1^*

^1^Group for Aquatic Microbial Ecology, Environmental Microbiology and Biotechnology, Faculty of Chemistry, University of Duisburg-Essen, Essen, Germany

^2^Regional University Center of Excellence in Environmental Industry, Szent István University, Gödöllő, Hungary

^3^Department of Environmental Protection and Safety, Szent István University, Gödöllő, Hungary

*to whom the correspondence should be addressed:

tancsics.andras@fh.szie.hu

alexander.probst@uni-due.de

**Content**

Description for File S1

Description for File S2

Description for File S3

Figure S1: Rank abundance curves of samples AER1, AER2 and AER3

Figure S2: Difference plots based on coverage of rpS3 sequences

**Description for File S1:** Phylogenetic tree in Newick format based on rpS3 amino acids sequences.

**Description for File S2:** Phylogenetic tree in Newick format of all recovered genomes using 16 ribosomal proteins.

**Description for File S3:** Phylogenetic tree in Newick format of Saccharibacteria based on 16S rRNA gene sequences.

**Figure S1. Rank abundance curves of samples AER1, AER2 and AER3 based on coverage (read-based mapping) of scaffolds carrying rpS3 sequences.**


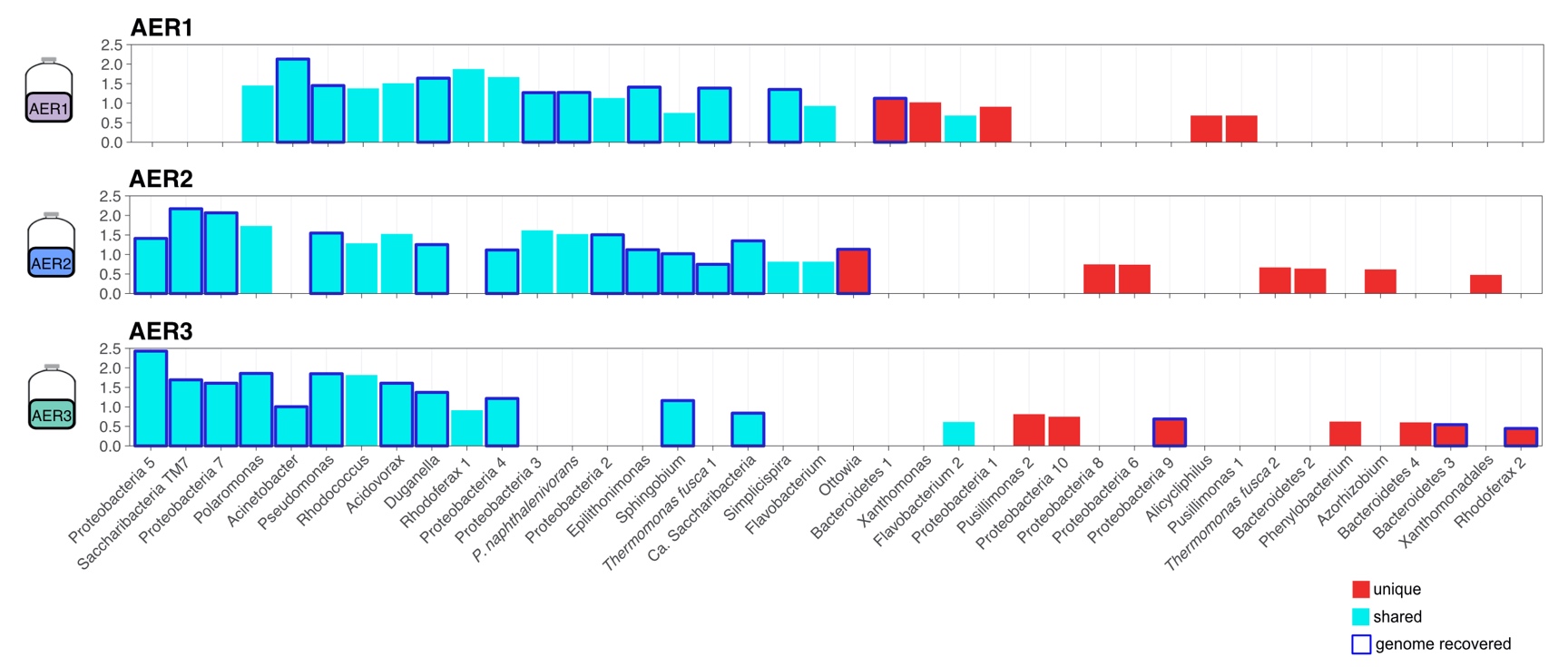


**Figure S2: Difference plots between samples AER1 and AER2 as well as AER1 and AER3. The plots are based on the read-based coverage of scaffolds carrying rpS3 sequences as presented in Fig. S1.**


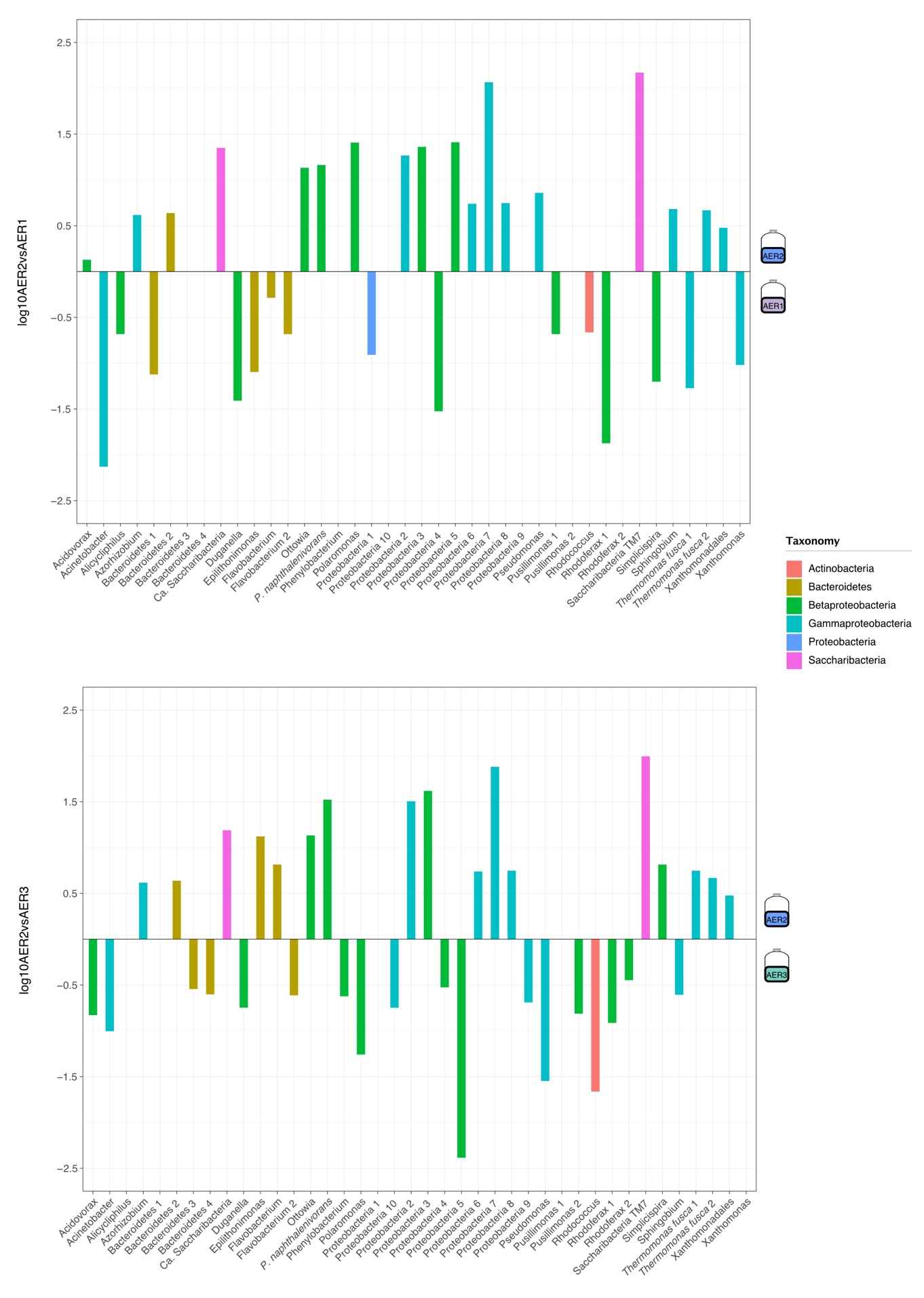

Supplement: Supplementary file 1 [file Data_Sheet_1.docx]
